# *Neisseria meningitidis* C:2b:P1.2,5 with Intermediate Resistance to Penicillin, Portugal

**DOI:** 10.3201/eid1003.030357

**Published:** 2004-03

**Authors:** Manuela Caniça, Ricardo Dias, Eugénia Ferreira, Meningococci Study Group

**Affiliations:** * National Institute of Health Dr. Ricardo Jorge, Lisbon, Portugal

**Keywords:** *Neisseria meningitides*, serogroup, serotype, serosubtype, penicillin susceptibility, phenotype C:2b:P1.2,5, emergence, Portugal

## Abstract

For 1 year, serogroup, serotype, serosubtype, and penicillin susceptibility of meningococci circulating in various regions in Portugal were evaluated. Most frequent phenotypes were B:4:P1.15 (13.4%) and C:2b:P1.2,5 (75.9%), which are also common in Spain. Overall, 27.5% of C:2b:P1.2,5 strains showed intermediate resistance to penicillin. Laboratory-based surveillance of meningococcal infection in Portugal provides important information to assess the adequacy of public health measures.

In Europe, infections caused by *Neisseria meningitidis* are associated with high rates of disease and death (*1*). Outbreaks of invasive meningococcal disease, including some cases of sudden death in some countries, suggest that vaccination may be necessary to reduce the incidence of this infection (*2*). Knowledge of the antigenic characteristics of *N. meningitidis* would help determine the most appropriate control strategies, which may vary from country to country.

Serogroups B and C are the most widespread, representing approximately 95% of cases of invasive meningococcal disease in Europe (*1*), and serogroup B is the most frequent cause of invasive meningococcal disease both in America and Europe (*3*). Serotypes and serosubtypes can be distinguished according to antigenic variants of the outer membrane proteins PorB and PorA (*4*).

In Portugal, the Compulsory Notifiable Diseases system is used, and meningococcal disease is a notifiable disease (*5*). The serogroup C conjugate vaccine was licensed in 2000, and vaccination has been voluntary since the third quarter of 2001. Laboratory analysis of serotypes and serosubtypes, not previously studied in meningococcal isolates from Portugal, would contribute to understanding meningococcal diversity and spread of meningococcal disease before the voluntary vaccination period.

We report a laboratory-based surveillance study of the *N. meningitidis* serogroups, serotypes, and serosubtypes in circulation in Portugal, as isolated from patients with cases of cultured-confirmed invasive infection. Susceptibility to penicillin was also evaluated.

## The Study

A total of 116 isolates of *N. meningitidis* were detected through a laboratory surveillance study; 27 hospitals from different regions of Portugal participated. The investigation was conducted for 12 consecutive months (September 2000 to August 2001) after a 2-month pilot study (July and August 2000) in six hospitals. The catchment population of the participating hospitals included approximately 7,400,000 residents (71% of the Portuguese population).

Isolates of *N. meningitidis* were directly submitted at –20ºC to the Antibiotic Resistance Unit in National Institute of Health Dr. Ricardo Jorge, Lisbon, as pure isolates. However, in some cases, only primary cultures performed at participant hospitals were sent, at 35ºC, and isolated in Antibiotic Resistance Unit. All strains were identified by standard methods (*6*).

The inclusion criteria for laboratory diagnosis were as follows: nonrepetitive and consecutive blood, cerebrospinal fluid (CSF) samples, or both in persons with symptoms compatible with invasive meningococcal disease. The strains from the pilot study were also included in the analysis. A case of invasive meningococcal disease was defined as disease in which *N. meningitidis* had been isolated by culture from two normally sterile sites (blood and CSF) in a resident of the surveillance area.

Serogroup was determined on 116 isolates by slide agglutination with the polyclonal specific rabbit antisera for A, C, X, Y, Z, W135, and 29E capsular polysaccharides of *N. meningitidis* and the monoclonal antibodies to determine serogroup B (Murex Diagnostic, Dartford, U.K.) (*6*). Serogroup findings by slide agglutination were checked by polymerase chain reaction with previously described primers (*7*).

For serotype and serosubtype analysis and susceptibility testing, 109 strains were available. Serotypes and subtypes were determined by an enzyme-linked immunosorbent assay method (*4*) by using monoclonal antibodies (RIVM, National Institute of Public Health and the Environment, the Netherlands). Serotype-specific reagents included 1, 4, 2a, 2b, 14, and 15; serosubtype-specific reagents included P1.1, P1.2, P1.4, P1.5, P1.6, P1.7, P1.9, P1.10, P1.12, P1.13, P1.14, P1.15, and P1.16.

The susceptibility to penicillin (Wyeth Lederle Portugal, Algés, Portugal) was assessed by determining MICs by using the agar dilution method with Mueller-Hinton agar supplemented with 5% sheep blood and incubated in 5% CO_2_ at 35°C for 24 h. *N. meningitidis* strains with an MIC of penicillin of <0.06 μg/mL were susceptible, strains with an MIC of penicillin of 0.12 μg/mL to 1 μg/mL had intermediate resistance to penicillin. *Escherichia coli* ATCC 25922 and *Staphylococcus aureus* ATCC 29213 were used as control strains. Production of β-lactamase was tested by nitrocefin (a chromogenic cephalosporin method).

The study sample included slightly more serogroup C (58/116, 50.0%) than serogroup B (55/116, 47.4%) isolates; the rest were serogroup W135 (3/116, 2.6%). The most frequent type and subtype of serogroup B was 4:P1.15 (13.4%) and of serogroup C was 2b:P1.2,5 (75.9%) (Table). Phenotype C:2b:P1.2,5 accounted for 37.6% of all strains, but no differences existed by age and region (data not shown). Most of the 41 strains with this phenotype were isolated from November 2000 through June 2001, with a peak from February 2001 to April 2001 (Figure). This peak contributed to the higher number of isolates obtained during this period.

**Table:** Distribution of 109 invasive meningococcal isolates by serogroup, serotype, serosubtype and susceptibility to penicillin

Serogroup (no. of strains)	Serotype (no. of strains)	Serotype: subtype^a^	No. of strains (%)	Penicillin susceptibility		No. of strains with the same phenotype in 1995–1999^b,c^	
				MIC (μg/mL)	S, IR^b^		
B (n = 52)	1 (n = 12)	1:NST	6 (11.6)	0.06	S		
		1:P1.13	2 (3.8)	0.06	S		
		1:P1.14	1 (1.9)	0.06	S		
		1:P1.15	1 (1.9)	0.25	IR		
		1:P1.4	1 (1.9)	0.06	S		
		1:P1.9	1 (1.9)	0.06	S		
	2b (n = 1)	2b:NST	1 (1.9)	0.06	S		
	4 (n = 16)	4:P1.2,5	2 (3.8)	0.06	S		
			1 (1.9)	0.125	IR		
		4:P1.4	3 (5.8)	0.06	S		
		4:P1.6	1 (1.9)	0.06	S		
		4:P1.14	1 (1.9)	0.06	S	1 (IR)	
		4:P1.15	5 (9.6)	0.06	S	1 (S)	
			2 (3.8)	0.125	IR		
		4:NST	1 (1.9)	0.06	S		
	14 (n = 2)	14:NST	1 (1.9)	0.06	S	3 (S)	
		14:P1.12	1 (1.9)	0.25	IR		
	15 (n = 6)	15:P1.7	1 (1.9)	0.06	S		
		15:P1.7,16	5 (9.6)	0.06	S	5 (S)	
	NT (n = 15)	NT:P1.2,5	1 (1.9)	0.06	S		
		NT:P1.6	1 (1.9)	0.06	S	2 (S)	
		NT:P1.7,16	1 (1.9)	0.06	S	1 (IR)	
		NT:P1.9	5 (9.6)	0.06	S	1 (S)	
		NT:P1.12	1 (1.9)	0.06	S		
		NT:P1.15	2 (3.8)	0.06	S	1 (S)	
			1 (1.9)	0.25	IR		
		NT:NST	3 (5.8)	0.06	S	1 (IR)	
C (n = 54)	2a (n = 2)	2a:P1.2,5	1 (1.9)	0.06	S	2 (S)	
		2a:P1.5	1 (1.9)	0.06	S		
	2b (n = 48)	2b:P1.2	2 (3.7)	0.06	S	1 (IR)	
			1 (1.9)	0.125	IR		
			1 (1.9)	0.25	IR		
		2b:P1.2,5	11 (20.4)	0.06	S	18 (3 S, 15 IR)	
			10 (18.5)	0.125	IR		
			18 (33.3)	0.25	IR		
			2 (3.7)	0.5	IR		
		2b:P1.5	1 (1.9)	0.06	S	1 (IR)	
		2b:NST	1 (1.9)	0.25	IR	1 (IR)	
			1 (1.9)				
	15 (n = 1)	15:NST	1 (1.9)	0.5	IR		
				0.06	S		
	NT (n = 3)	NT:P1.2,5	1 (1.9)	0.06	S		
		NT:P1.5	1 (1.9)	0.06	S		
		NT:NST	1 (1.9)	0.06	S		
W135 (n = 3)	2a (n = 3)	2a:P1.2,5	2 (66.7)	0.06	S	4 (3S, 1IR)	
			1 (33.3)	0.125	IR		

^a^NT, non-serotypable; NST, non-serosubtypeable. ^b^S, susceptible; IR, intermediate resistance. ^c^Phenotypes that already existed in a strain collection isolated in nine Portuguese hospitals from January 1995 to December 1999 (n = 54) (8). Phenotypes present in the earlier collection not found in our study (unpub. data) are: B:2b:P1.5, B:4:P1.7, B:15:P1.9, B:15:P1.13, B:15:NST, B:NT:P1.14, B:NT:P1.16, C:2a:P1.14, C:2a:NST, C:4:P1.12; W135:NT:NST (n = 1 for each phenotype).

**Figure:**
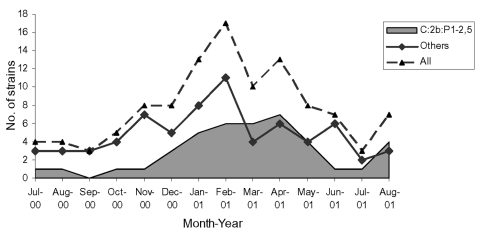
Distribution of *Neisseria meningitidis* collected in Portugal from July 2000 to August 2001 and phenotype C:2b:P1.2,5.

Strains with intermediate resistance to penicillin from serogroup B had the following serotypes and subtypes: 4:P1.15 (two strains) and one strain of each 1:P1.15, 4:P1.2,5, 14:P1.12 and NT:P1.15. Strains with intermediate resistance to penicillin from serogroup C had the following serotypes and subtypes: 2b:P1.2,5 (30 strains) and 2b:P1.2 (2 strains) and 2b:NST (2 strains). One strain from the phenotype W135:2a:P1.2,5 also showed intermediate resistance to penicillin (Table).

## Conclusions

This study, covering approximately 71% of the Portuguese population, shows that serogroups B and C are dominant in Portugal, as they are elsewhere in Europe (*1*). Meningococcal disease in our study followed the usual European pattern (*1*), including the seasonal peak in winter and the age distribution, with children <5 years of age being the most affected group (data not shown).

B:15:P1.7,16 and B:4:P1.4 were the fourth and fifth most frequent serotype and subtypes of serogroup B in our sample, respectively, but were the major phenotypes of invasive meningococci in other European countries in 1999 and 2000, followed by B:4:P1.15, B:1:P1.14 and B:4:P1:10, in descending order (*1*,*9*). However, the most frequent serotype and subtype of serogroup B in this study was 4:P1.15 (13.4%), as in Spain (*1*). Spain was the only European country to report phenotype B:4:P1.15 from 1993 to 1996 (*9*), but it was found in the Czech Republic, Slovakia, and Malta in 1999 through 2000 (*1*).

Between serogroup C strains, those with serotype and subtype 2b:P1.2,5 (75.9%) were the most frequent, followed by 2b:P1.2 (7.5%) (1,9). In Europe, in 1999 through 2000, the prevalent phenotypes for strains of serogroup C were C:2a:P1.5 and C:2a:P1.2,5, followed by C:2b:P1.2,5, C:2b:P1.2, and C:2a:P1.2 (*1*).

The apparent endemicity of *N. meningitidis* C:2b:P1.2,5 in Portugal (Figure) (*8*), as in Spain, suggests that the features of the Iberian Peninsula are favorable for this phenotype. Invasive meningococcal disease may be caused by host factors more than bacterial determinants (*10*,*11*). Molecular analysis may be able to indicate whether the phenotype 2b:P1.2,5 is a variant of clones spreading in other countries or even a variant of previously identified clones in Portugal. Monitoring the incidence of this phenotype and its apparent emergence from 1995 (33.3%) to 2001 (75.9%) (Table) (*8*) is important.

The three strains of serogroup W135 in this study were all serotype and subtype 2a:P1.2,5, the predominant clone in Europe (*1*). This serotype was the cause of an outbreak in 2000, after the Hajj pilgrimage to Mecca (*12*). We have no information about contact between patients and Hajj pilgrims or the European outbreak. However, the main serotype and subtype in serogroup W135 in Portugal previously (between 1995 and 1999) was also 2a:P1.2,5 (Table).

The emergence of serogroup C, including numerous isolates with intermediate resistance to penicillin (*13*,*14*), leads us to emphasize the importance of the prophylaxis and the need for cost-benefit studies to control meningococcal disease. Resistance to penicillin can impede treatment of invasive disease, making surveillance of this resistance important. Strains of phenotypes B:4:P1.15 and C:2b:P1.2,5 most frequently had intermediate resistance to penicillin, and C:2b:P1.2,5 strains had the highest MICs of penicillin (all isolates with MIC of 0.5 μg/mL and 95% with MIC of 0.25 μg/mL). In Spain in the 1980s, types 4:P1.15 (serogroup B) and 2b (serogroup C) were also found in the main meningococci with intermediate resistance to penicillin (*14*).

In conclusion, continued serogrouping of meningococcal strains would be valuable because possible antigen variations caused by capsular switching (*15*) can occur after the period of this study, especially because of the voluntary vaccination program available in Portugal. Meningococci use this mechanism to escape control by vaccines or the natural immune protection. Consequently, the pattern of phenotypes could be subject to major changes, and adjustment to new circumstances will be needed. Antigenic characterization has contributed to the understanding of meningococcal diversity and spread of meningococcal disease. Trends were detected epidemiologically, and serotyping provided further information, which can also contribute to establishing strategies for developing a universal serogroup B vaccine. Molecular typing of *N. meningitidis* is also needed in Portugal to follow evolutionary changes in the bacteria and to elucidate clonal relationships between isolates.

Our results also demonstrate the importance of monitoring susceptibility to antimicrobial drugs and antigenic characteristics of meningococci; in Portugal, the prevalent phenotype C:2b:P1.2,5 is of particular concern.
